# Identification of potential blood biomarkers for early diagnosis of Alzheimer’s disease through RNA sequencing analysis

**DOI:** 10.1186/s13195-020-00654-x

**Published:** 2020-07-16

**Authors:** Daichi Shigemizu, Taiki Mori, Shintaro Akiyama, Sayuri Higaki, Hiroshi Watanabe, Takashi Sakurai, Shumpei Niida, Kouichi Ozaki

**Affiliations:** 1 Medical Genome Center, National Center for Geriatrics and Gerontology, 7-430 Morioka-cho, Obu, 474-8511 Aichi Japan; 2 Department of Medical Science Mathematics, Medical Research Institute, Tokyo Medical and Dental University (TMDU), Tokyo, 113-8510 Japan; 3 RIKEN Center for Integrative Medical Sciences, Yokohama, 230-0045 Kanagawa Japan; 4 The Center for Comprehensive Care and Research on Memory Disorders, National Center for Geriatrics and Gerontology, Obu, 474-8511 Aichi Japan; 5 Department of Cognitive and Behavioral Science, Nagoya University Graduate School of Medicine, Nagoya, 466-8550 Aichi Japan

**Keywords:** Alzheimer’s disease, RNA sequencing, Biomarkers for early diagnosis

## Abstract

**Background:**

With demographic shifts toward older populations, the number of people with dementia is steadily increasing. Alzheimer’s disease (AD) is the most common cause of dementia, and no curative treatment is available. The current best strategy is to delay disease progression and to practice early intervention to reduce the number of patients that ultimately develop AD. Therefore, promising novel biomarkers for early diagnosis are urgently required.

**Methods:**

To identify blood-based biomarkers for early diagnosis of AD, we performed RNA sequencing (RNA-seq) analysis of 610 blood samples, representing 271 patients with AD, 91 cognitively normal (CN) adults, and 248 subjects with mild cognitive impairment (MCI). We first estimated cell-type proportions among AD, MCI, and CN samples from the bulk RNA-seq data using CIBERSORT and then examined the differentially expressed genes (DEGs) between AD and CN samples. To gain further insight into the biological functions of the DEGs, we performed gene set enrichment analysis (GSEA) and network-based meta-analysis.

**Results:**

In the cell-type distribution analysis, we found a significant association between the proportion of neutrophils and AD prognosis at a false discovery rate (FDR) < 0.05. Furthermore, a similar trend emerged in the results of routine blood tests from a large number of samples (*n* = 3,099: AD, 1,605; MCI, 994; CN, 500). In addition, GSEA and network-based meta-analysis based on DEGs between AD and CN samples revealed functional modules and important hub genes associated with the pathogenesis of AD. The risk prediction model constructed by using the proportion of neutrophils and the most important hub genes (*EEF2* and *RPL7*) achieved a high AUC of 0.878 in a validation cohort; when further applied to a prospective cohort, the model achieved a high accuracy of 0.727.

**Conclusions:**

Our model was demonstrated to be effective in prospective AD risk prediction. These findings indicate the discovery of potential biomarkers for early diagnosis of AD, and their further improvement may lead to future practical clinical use.

## Background

With demographic shifts toward older populations, the number of people with dementia is steadily increasing. The total number of people with dementia worldwide has been estimated to be 75 million by 2030 and 135 million by 2050 [1]. Since there is no treatment or prevention for AD, the current best strategy is to delay disease progression and to practice early intervention to reduce the number of patients that ultimately develop AD [2]. Therefore, promising novel biomarkers for early diagnosis are urgently required [3, 4].

Alzheimer’s disease (AD) is the most common cause of dementia, accounting for 60 to 80% of dementia cases [5]. Genome-wide association studies (GWAS) have identified several genetic factors that contribute to AD risk [6–8]. However, the cause of the disease still remains to be elucidated. The current AD diagnosis is generally based on assessing patients’ cognitive function. These examinations are not performed routinely, because they are time-consuming and the results largely depend on the physician’s experience [9, 10]. Alternatively, cerebrospinal fluid (CSF) biomarkers, including amyloid-beta 1–42 (Aβ_1-42_), total tau (T-tau), and phosphorylated tau 181 (P-tau_181_) [11, 12], and positron emission tomography (PET) imaging scans [13–15] are effective for AD diagnosis, but because of the highly invasive nature of CSF collection and high cost of PET, using these biomarkers as part of a general physical examination to facilitate early diagnosis and therapeutic intervention remains challenging.

Compared with CSF biomarkers and PET imaging scans, blood-based biomarkers are attractive as affordable alternatives for the diagnosis of AD. Mattsson et al. recently reported that plasma neurofilament light level (NfL) has the potential to be a noninvasive biomarker to monitor neurodegeneration in AD [16]. Janelidze et al. reported that plasma P-tau_181_ is a noninvasive diagnostic and prognostic biomarker of AD [17]. One of the most powerful tools for detecting those biomarkers, whole RNA sequencing (RNA-seq) of human peripheral blood mononuclear cells (PBMCs) by using a next-generation sequencer, is widely applied and supports comprehensive analysis of the entire transcriptome [18–20]. The most important application of the RNA-seq data analysis is the identification of differentially expressed genes (DEGs) [21–23]. Systems biology analyses using DEGs reveal key functional modules and important hub genes associated with the pathogenesis of diseases (e.g., Gene Ontology [GO] [24, 25], Kyoto Encyclopedia of Genes and Genomes [KEGG] biological pathways [26, 27]). However, to our knowledge, no previous studies have involved comprehensive RNA-seq analysis of a large number of AD samples and applied an mRNA-based risk prediction model to a prospective cohort.

Here, we performed large-scale RNA-seq transcriptome analyses on a large number of AD samples to detect potential blood-based biomarkers for earlier diagnosis of AD. To this end, we used the RNA-seq data to evaluate cell-type composition among samples from subjects with AD, mild cognitive impairment (MCI), and normal cognitive function (CN) and to compare DEGs between AD and CN samples. Subsequent gene set enrichment analyses (GSEA) and network-based meta-analysis using the DEGs revealed new potential biomarkers for AD diagnosis. The risk prediction model using those potential biomarkers achieved a high AUC in a validation cohort and effectively determined AD risk in a prospective cohort. We believe that, once optimized, these new potential biomarkers will be of practical clinical use in the early diagnosis of AD.

## Methods

### Sample collection

All of the 610 subjects whose blood samples were evaluated for mRNA expression and their associated clinical data were obtained from the National Center for Geriatrics and Gerontology (NCGG) Biobank, which collects human biomaterials and data for geriatrics research. Of them, 271 subjects were AD patients, 91 subjects were elderly CN controls, and 248 patients had mild cognitive impairment (MCI). All of the subjects were 60 years or older (Supplementary Table S1). The AD and MCI subjects were diagnosed with probable or possible AD according to the criteria of the National Institute on Aging Alzheimer’s Association workgroups [9, 10]. Patients with probable AD were used as AD subjects in this study. The CN subjects had subjective cognitive abnormalities but normal cognition on a neuropsychological assessment, which included a comprehensive neuropsychological test, Mini-Mental State Examination (MMSE) score > 27. All of the 3,099 subjects (1,605 ADs, 994 MCIs, and 500 CNs) with the proportion of neutrophils measured in routine blood tests were also obtained from the NCGG Biobank. All of these subjects were also ≥ 60 years in age (Supplementary Table S2).

### cDNA library preparation and RNA sequencing

Buffy coat samples were isolated from the whole blood according to the standard operating procedure of NCGG Biobank [28]. Buffy coat fractions containing leukocytes were separated by centrifugation (3,500 rpm, 5 min, RT) and were frozen for further use. Total RNAs in buffy coat samples were isolated using the miRNeasy Mini Kit (Qiagen, Hilden, Germany) according to the manufacturer’s instructions with slight modification. TRIzol LS reagent (1 mL) (Thermo Fisher Scientific, MA, USA) and 1-bromo-3-chloropropane (100 μL) (Tokyo Chemical Industry, Tokyo, Japan) were added to each sample. Samples were mixed thoroughly by shaking for more than 30 s and incubated at room temperature for 3 min. Phase separation was performed by centrifugation at 15,000*g* at 4 °C for 15 min. The upper aqueous phase was collected and loaded into the miRNeasy mini-column. After washing, total RNAs were extracted by RNase free water (50 μL). Only high-quality samples with an RNA integrity number (RIN) value ≥ 6.0 were used to construct the sequencing library (Supplementary Table S1). Sequencing libraries were prepared by using 500 μg of total RNA for each sample with Illumina TruSeq Stranded Total RNA with Ribo-Zero Globin and IDT for Illumina-TruSeq UD Indexes according to the manufacturer’s instructions (Illumina, San Diego, CA). The libraries were subsequently sequenced by using Illumina NovaSeq6000 platform with paired-end reads of 151 bp according to the manufacturer’s instructions.

### RNA sequencing data analysis

The quality of the read sequences (fastq files) was assessed by using FastQC (version 0.11.7). The low-quality reads (< Q20) and trimmed reads with adaptor sequences (shorter than 50 bp) were discarded by using Cutadapt (version 1.16). The remaining clean sequenced reads were mapped to the human reference genome (GRCh37) by using STAR [29] (2-pass option, version 2.5.2b). By using the featureCounts program [30] from the subread package (version 1.6.6), read counts for each gene were calculated to generate expression levels. Outlier read counts (i.e., the top and bottom 5% of read counts for each gene) were replaced as the maximum and minimum of the remaining effectives, respectively. The read counts from each sample were then combined into a count file, on which differential expression analysis was performed by using edgeR [31] (version 3.18.1). Genes with a threshold CPM (counts per million reads mapped) > 1 in more than onefourth of all sequenced samples were used for further analysis. The caclNormFactorsfunction in edgeR [31] was used to obtain a trimmed mean of *M* value normalization factors to account for library sizes. Dispersion was calculated by using the estimateCommonDisp and estimateTagwiseDisp functions in edgeR [31]. The exactTest function in edgeR [31] was applied to obtain DEGs between AD and CN samples.

### Proportions of immune cell types according to bulk RNA sequencing data

After RNA-seq reads were aligned to the human reference genome by using STAR [29], RSEM [32] (version 1.3.0) was used to quantify transcripts per million (TPM), which were suitable for use with CIBERSORT [33] (version 1.0.1). While CIBERSORT estimated the proportions of 22 immune cell types, we recategorized these 22 cell types into 12 major cell types by summing the proportions as appropriate. The 12 cell types we evaluated were (1) B cells (naive and memory), (2) plasma cells, (3) CD8^+^ T cells, (4) CD4^+^ T cells (CD4^+^ T cells naive, memory resting, and memory activated; T cells follicular helper; and T cells regulatory), (5) γδ T cells, (6) NK cells (resting and activated), (7) monocytes, (8) macrophages (M0, M1, and M2), (9) dendritic cells (resting and activated), (10) mast cells (resting and activated), (11) eosinophils, and (12) neutrophils.

### In silico biological and functional analysis

Gene Ontology (GO) [24, 25] classification, which is comprised of three major categories—biological process, cellular component, and molecular function—is useful for uncovering the functions of genes of interest. The DAVID [25, 34] (version 6.8) gene functional classification tool (https://david.ncifcrf.gov) was used to generate annotations. DAVID was applied to a list of differentially expressed genes with FDR < 0.05 and fold change > 1.2, and statistically significant GO terms and KEGG biological pathways were identified. Statistically significant GO terms were further expressed as a *z*-score (the number of upregulated genes minus the number of downregulated genes divided by the square root of the count) and presented in a circular visualization by using the *GOplot* package (version 1.0.2) in R [35].

### Network-based meta-analysis

Network-based analysis was performed by using NetworkAnalyst [36] with the STRING Interactome database [37], which provides comprehensive information regarding interactions between proteins, including prediction and experimental interaction data. The confidence cutoff score was set to 700. The protein–protein interaction (PPI) network was constructed by using zero-order interaction network analysis (direct interaction only) and graphically generated by using Cytoscape v3.7.1 (http://www.cytoscape.org/) [38].

### Risk prediction model construction

RNA-seq data were split: two-thirds were used for a training data set and one-third for a test data set. Using the training data, we constructed risk prediction models based on clinical information (age, sex, and *APOE* ε_4_ genotypes), the proportion of neutrophils, and the top-ranked *p* hub genes using a random forest classifier. The top-ranked *p* hub genes were then selected stepwise (*p* = 1, 2, … 10). The optimal hyper-parameters in the training data were determined by using 10-fold cross-validation. The adjusted model was then evaluated on the test data, which were completely independent of the training data, by using AUC as the discriminative accuracy of the risk prediction model. The method used in this study was implemented through the *caret* package (version 6.0.76) in R (https://www.r-project.org/).

### qRT-PCR validation of gene expression

cDNA was synthesized by using a PrimeScriptII 1st Strand cDNA Synthesis Kit (Takara Bio, Shiga, Japan). Quantitative RT-PCR (qRT-PCR) analysis was performed by using customized TaqMan gene expression assays (Applied Biosystems, Waltham, MA) and the Quantstudio7 Flex Real-Time PCR System (Thermo Fisher, Waltham, MA). The following commercially available TaqMan gene expression assays were used: *EEF2* (Hs00157330_m1), *RPL7* (Hs02596927_g1), *LDHB* (Hs00929956_m1), *NR1D2* (Hs00233309_m1), *PDK4* (Hs01037712_m1), *TRIOBP* (Hs00980819_m1), *TAS2R39* (Hs00603443_s1), *BASP1* (Hs00932356_s1), and *ACTB* (Hs01060665_g1). The qRT-PCR conditions were as follows: one cycle of 50 °C for 2 min and 95 °C for 20 s followed by 40 cycles of 95 °C for 1 s, 60 °C for 20 s, and 72 °C for 30 s. Each gene was assayed in duplicate. *ACTB* was pre-selected as a reference gene for normalization of target gene expression levels. Gene expression levels from qRT-PCR were calculated relative to the reference gene *ACTB* using the semi-quantitative method [39]. The gene expressions were obtained for 10 AD and 10 CN randomly selected samples. The log2 fold change (logFC) was obtained from the average values of the gene expressions.

## Results

### RNA sequencing data

A total of 610 samples, comprising 271 AD, 248 MCI, and 91 CN samples, were enrolled in this study (Table 1). Using a high-throughput next-generation system to perform RNA sequencing (RNA-seq) analysis, we obtained an average of 44.3, 47.3, and 43.9 million raw read sequences from the AD, MCI, and CN samples, respectively, of which 99.6%, 99.5%, and 99.6% were high-quality (i.e., > Q20) read sequences. After low-quality read sequences were discarded and reads with adaptor sequences were trimmed, 43.8, 47.3, and 43.2 million reads of cleaned data remained for the AD, MCI, and CN samples, respectively, of which 82.7%, 82.1%, and 82.1% uniquely mapped to the human reference genome (GRCh37) (Supplementary Table S3).

**Table 1:** Summary of characteristics for AD, MCI, and CN samples

Characteristic	AD	MCI	CN
Sample number	271	248	91
Male:female	1:2.15	1:1.30	1:0.82
Age (mean ± 1 S.D.)	79.55 ± 5.83	77.37 ± 6.12	71.29 ± 5.07
MMSE (mean ± S.D.)	18.09 ± 4.49	24.54 ± 2.98	29.32 ± 0.94
*APOE* genotypes	E2/2 = 2, E3/2 = 14, E3/3 = 148, E4/2 = 3, E4/3 = 88, E4/4 = 16	E2/2 = 1, E3/2 = 11, E3/3 = 163, E4/2 = 1, E4/3 = 60, E4/4 = 12	E3/2 = 5, E3/3 = 73, E4/3 = 12, E4/4 = 1

*MMSE* Mini-Mental State Examination (a comprehensive neuropsychological test)

### Comparison of cell-type distribution among AD, MCI, and CN samples

**Fig. 1:**
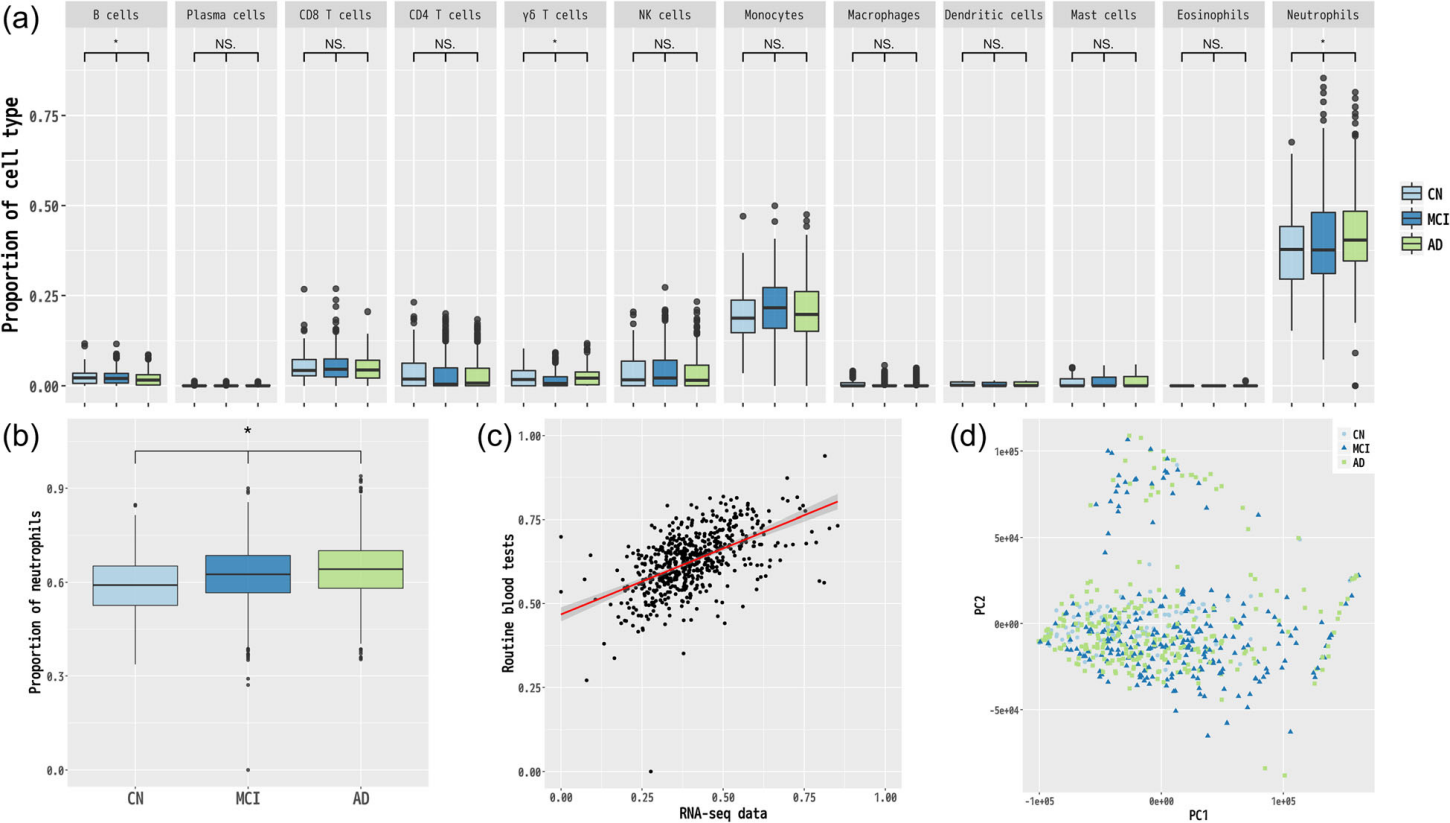
Proportions of the 12 major immune cell types among samples from patients with AD, MCI, and CN. **a** Comparison of cell types among AD, MCI, and CN samples (*FDR < 0.05, Jonckheere–Terpstra trend test). **b** Proportion of neutrophils in AD, MCI, and CN samples according to routine blood tests. **c** Correlation between the proportions of neutrophils estimated from RNA-seq data and that from routine blood tests. **d** PCA using RNA-seq data

To detect blood-based biomarkers, we first used the bulk RNA-seq data to compare cell-type distribution among AD, MCI, and CN samples. Specifically, CIBERSORT [33] estimated the relative proportions (as transcripts per million [TPM]) of 12 major types of immune cells (i.e., B cells, plasma cells, CD8^+^ T cells, CD4^+^ T cells, γδ T cells, NK cells, monocytes, macrophages, dendritic cells, mast cells, eosinophils, and neutrophils) in each sample. We used the Jonckheere–Terpstra trend test to identify a statistically significant increase or decrease in cell-type proportion among AD, MCI, and CN samples. Accordingly, the proportion of neutrophils was significantly increased in AD prognosis at an FDR < 0.05 (neutrophils, 0.007; Fig. 1a and Supplementary Table S1). The proportions of B cells and *γδ* T cells also showed significant differences in AD prognosis at an FDR < 0.05 (B cells, 0.019; *γδ* T cells, 0.007; Fig. 1a and Supplementary Table S1), but these proportions were very low in all samples and too difficult to determine if they were truly associated with the AD prognosis.

To further investigate the association between an increased neutrophil count and AD prognosis, we used a larger number of samples (*n* = 3,099: AD, 1,605; MCI, 994; and CN, 500) to examine the neutrophil population determined through routine blood tests. Interestingly, these data sets obtained by using routine blood tests revealed the same increase in the neutrophil proportion as the RNA-seq data (*P* = 0.002, Jonckheere–Terpstra trend test; Fig. 1b and Supplementary Table S2). Therefore, these results provided strong evidence that an increased neutrophil proportion might be useful as a blood-based biomarker for the diagnosis of AD. The proportion of neutrophils estimated from RNA-seq data was positively correlated with that calculated through routine blood tests (254 ADs, 232 MCIs, and 85 CNs; Pearson *r* = 0.56, *P* < 0.01, Fig. 1c). We also performed a principal component analysis with RNA-seq data of the three groups, but we could not observe the significant difference among the three (Fig. 1d).

### Detection of DEGs

**Fig. 2:**
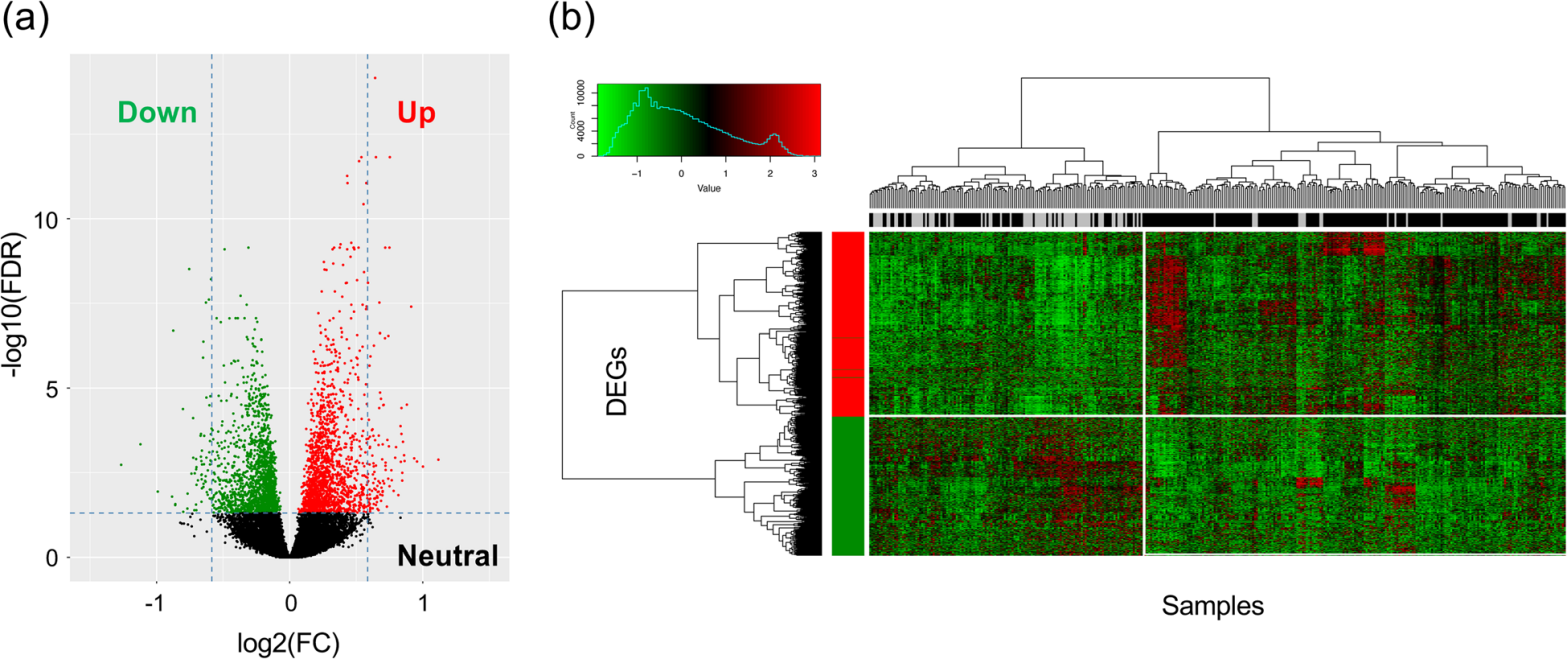
Distribution of differentially expressed genes (DEGs). **a** Each point represents a gene. Green and red dots represent downregulated and upregulated DEGs, respectively. **b** Hieratical clustering of DEGs and samples by using the trimmed mean of M-values normalization factors. The horizontal and vertical axes represent the samples (AD, black; CN, gray) and DEGs (red, upregulated; green, downregulated), respectively

Focusing on the 19,699 genes with a threshold of > 1 CPM (counts per million reads mapped) in more than one-fourth of all sequenced samples, we next examined the DEGs in AD and CN samples. A total of 846 statistically significant DEGs (i.e., FDR < 0.05 and fold change > 1.2) with Entrez gene IDs were identified, of which 480 genes were upregulated and 366 were downregulated in the AD samples (Fig. 2a and Supplementary Table S4). In addition, a heatmap of DEGs using the trimmed mean of *M* value normalization factors showed that the expression profiles of the AD and CN samples clustered separately (Fig. 2b).

### Biological and functional analysis

**Fig. 3:**
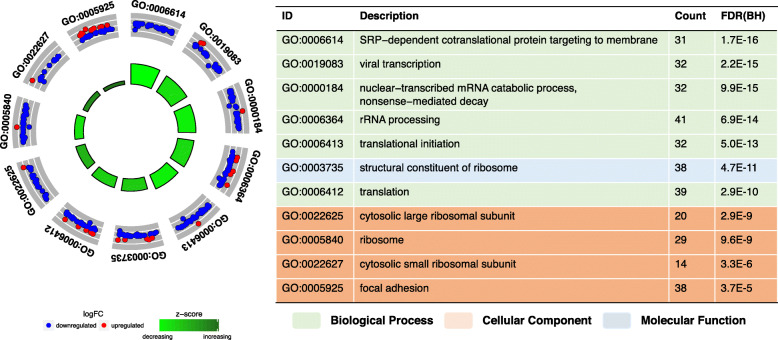
Gene set enrichment analysis using DEGs. Statistically significant Gene Ontology terms with a false discovery rate < 0.05

To gain further insight into the biological functions of the DEGs, we performed a gene set enrichment analysis (GSEA) using the DAVID (version 6.8) gene functional classification tool (https://david.ncifcrf.gov) [25, 34]. As a result, the DEGs were enriched in 11 GO terms (6 biological processes, 4 cellular components, and 1 molecular function) and one KEGG biological pathway (hsa03010: ribosome), with a significance level set at FDR < 0.05. The enrichment levels of those GO terms are presented in circular visualization (Fig. 3). The GO terms were enriched in many downregulated genes (Fig. 3), and most of them involved ribosomal subunits: 19 RPL genes (*RPL3*, *RPL5*, *RPL6*, *RPL7*, *RPL9*, *RPL10A*, *RPL11*, *RPL18*, *RPL19*, *RPL21*, *RPL22*, *RPL23*, *RPL23A*, *RPL26*, *RPL27*, *RPL29*, *RPL32*, *RPL35*, and *RPL36AL*), 12 RPS genes (*RPS3*, *RPS3A*, *RPS4Y1*, *RPS5*, *RPS6*, *RPS8*, *RPS11*, *RPS12*, *RPS14*, *RPS18*, *RPS24*, and *RPS29*), and 3 MRP genes (*MRPS5*, *MRPL16*, and *MRPL47*).

### Network-based meta-analysis

**Fig. 4:**
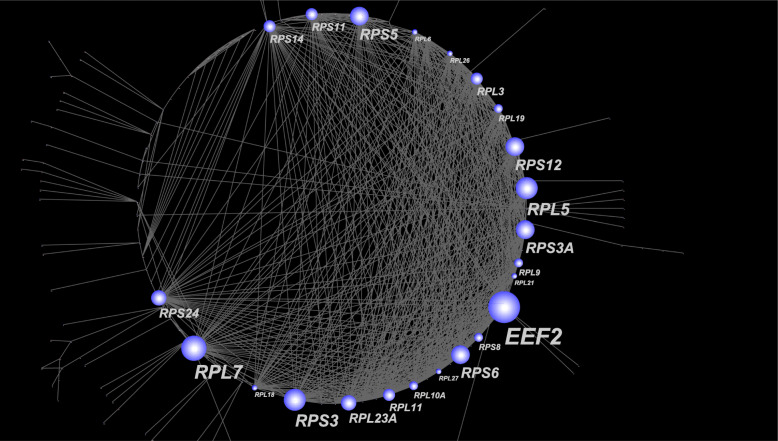
Network-based meta-analysis using DEGs. A protein–protein interaction network detected in DEGs

In addition to GSEA, we performed a protein–protein interaction (PPI) network analysis based on the DEGs by using NetworkAnalyst [36] (http://www.networkanalyst.ca) with the STRING Interactome database [37]. As a result, we obtained a PPI network comprising 4,164 nodes and 11,886 edges. To prune the network to a more manageable size, we conducted a zero-order interaction network analysis and detected a network containing 161 nodes and 700 edges (Fig. 4). The most highly ranked hub genes were recognized in terms of network topology measures of degree (DC) and betweenness of centrality (BC). The top-ranked 10 hub genes were *EEF2* (eukaryotic elongation factor 2, DC = 38, BC = 883.9, FC = 1.22, FDR = 0.048) and 9 ribosomal proteins: 3 RPL genes (*RPL5*, *RPL7*, and *RPL23A*) and 6 RPS genes (*RPS3*, *RPS3A*, *RPS5*, *RPS6*, *RPS12*, and *RPS24*) (Table 2). Many of the identified genes were common to those obtained through GSEA.

**Table 2:** Top-ranked 10 hub genes detected in the network-based meta-analysis using DEGs

Gene name	DC	BC	FC	FDR
*EEF2*	38	883.9	1.22	4.80 × 10^−3^
*RPL7*	36	350.3	1.51	1.75 × 10^−5^
*RPL5*	35	4567.0	1.23	2.07 × 10^−5^
*RPS3*	35	1599.7	1.38	1.89 × 10^−4^
*RPS5*	34	741.0	1.35	0.024
*RPS12*	34	427.3	1.44	5.20 × 10^−4^
*RPS3A*	34	23.1	1.31	1.10 × 10^−3^
*RPS6*	34	23.1	1.23	3.31 × 10^−5^
*RPL23A*	33	48.1	1.27	8.50 × 10^−3^
*RPS24*	33	22.0	1.23	8.00 × 10^−4^

The most highly ranked hub genes in terms of network topology measures of degree (DC) and betweenness of centrality (BC)

*FC* fold change, *FDR* false discovery rate

### Validation of potential biomarkers of AD in blood

**Fig. 5:**
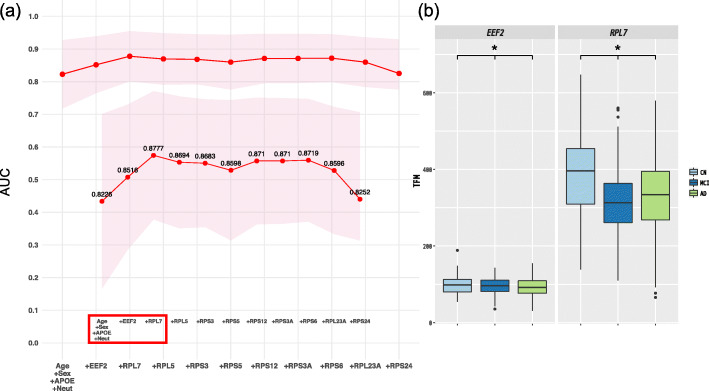
Potential biomarkers of AD in the blood by using the most important hub genes. **a** Identification of the most important hub genes by using a random forest classifier. Neut, neutrophils. **b** Expression of two hub genes (*EEF2* and *RPL7*) among AD, MCI and CN samples

We examined whether many of the top-ranked hub genes could be potential blood biomarkers for AD. For this purpose, two-thirds of all samples were used as a training data set (240 samples: 180 ADs and 60 CNs), and the remaining one-third was used as a test data set (122 samples: 91 ADs and 31 CNs). The top-ranked *p* hub genes were selected stepwise. A risk prediction model was constructed by using clinical information (age, sex, and *APOE* ε_4_ genotypes), the proportion of neutrophils, and the top-ranked *p* hub genes with a random forest classifier using the training data. The adjusted model was then evaluated on the independent test data by using the area under the receiver operating characteristic curve (AUC). The best model achieved an AUC of 0.878 (95% CI 0.801–0.955, sensitivity = 0.945, specificity = 0.710, Supplementary Fig. S1a) in the test data when two top-ranked hub genes (*EEF2* and *RPL7*, Fig. 5a) were used. The highest variable importance was age (MeanDecreaseGini = 30.97; *RPL7*, 20.63; Neut, 14.23; *EEF2*, 14.20; *APOE* ε_4_ genotypes, 5.05; sex, 3.15). The best model had a superior AUC to the model using only clinical information (Fig. 5a). Furthermore, the expression of two hub genes, *EEF2* and *RPL7*, were associated with a significant decrease and increase in AD prognosis, respectively (*P* = 0.015 in *EEF2*, *P* = 0.032 in *RPL7*, Jonckheere–Terpstra trend test, Fig. 5b and Supplementary Table S5). These results suggested that these two hub genes could serve as potential diagnostic blood biomarkers of AD. In a similar way, risk prediction models were constructed by using clinical features (age, sex, and *APOE* ε_4_ genotypes), the proportion of neutrophils, and the top-ranked two hub genes with a random forest classifier using the training data. The adjusted models were then evaluated on the independent test data for a MCI and CN set and a MCI and AD set. The best models achieved an AUC of 0.683 (95% CI = 0.559–0.807, sensitivity = 0.744, specificity = 0.633, Supplementary Fig. S1b) and an AUC of 0.645 (95% CI 0.562–0.728, sensitivity = 0.622, specificity = 0.671, Supplementary Fig. S1c) for the MCI and CN set and the MCI and AD set in the test data, respectively.

### Validation in a prospective cohort

**Fig. 6:**
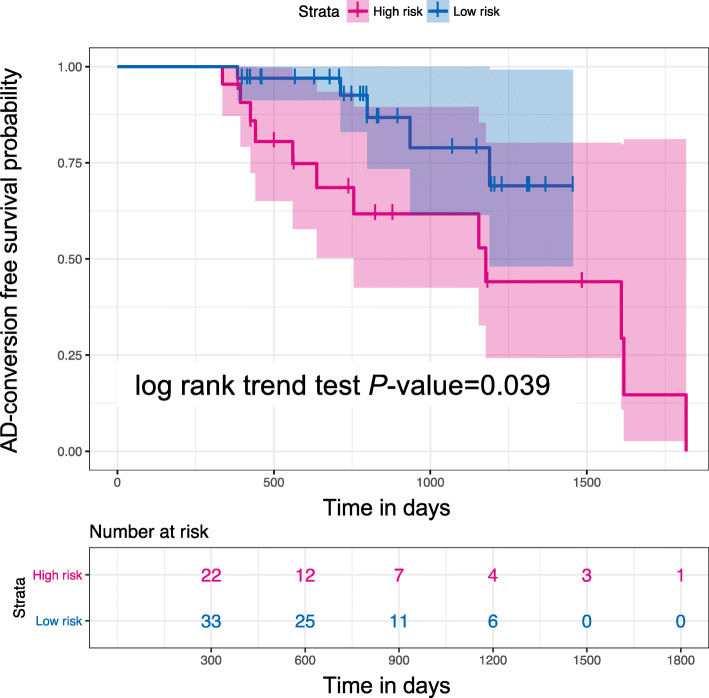
Validation of potential biomarkers by using a prospective cohort

We measured mRNA expression in 248 MCI samples. Of them, 55 MCI samples were obtained from the prospective data; 17 patients who contributed samples progressed to AD, whereas 38 of the patients corresponding to these samples have not yet been diagnosed with bona fide AD after at least 1 year. Our risk prediction model based on clinical information (age, sex, and *APOE* ε_4_ genotypes) and three potential biomarkers we obtained (i.e., the proportion of neutrophils, *EEF2*, and *RPL7*) was applied to the prospective data. Because our prediction model provides a probability of AD conversion for each MCI sample, we set the MCIs at the probability of > 0.9 for conversion to AD. Survival probabilities were calculated by using the Kaplan–Meier method in the *survival* package (version 2.41.3) for the statistical software R. Our risk prediction model significantly classified the MCI samples into two categories (high and low risk). The Kaplan–Meier curves showed improved outcome for AD conversion-free survival (Fig. 6, log rank trend test = 0.039), which achieved a high accuracy of 0.727 on the prospective cohort (sensitivity = 0.706, specificity = 0.737). Our present model predicted that 33 samples would not covert to AD, of which 5 did convert (negative predictive value (NPV) = 0.848). Although this clear classification of samples might be helpful for future practical use in healthcare, we would have to follow those samples to improve the further predictive value.

### Verification of quantitative RT-PCR assay

**Fig. 7:**
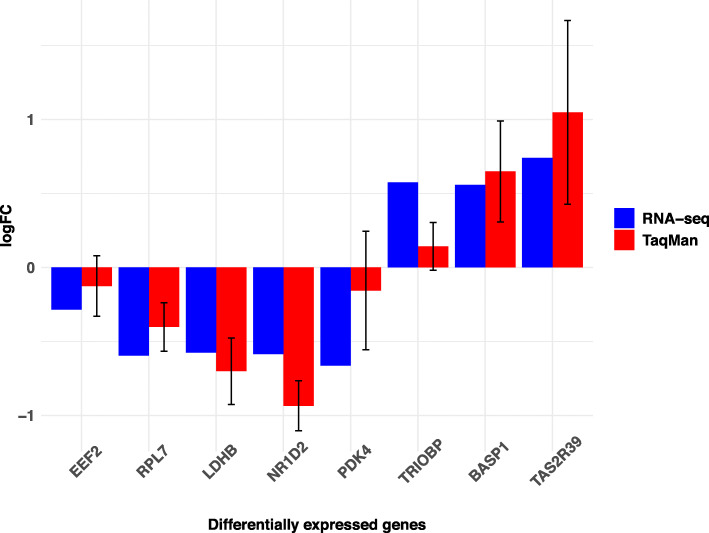
qRT-PCR verification of DEGs detected by RNA-seq. Fold change values obtained from RNA-seq data: *TRIOBP*, *TAS2R39*, and *BASP1* were upregulated, and *EEF2*, *RPL7*, *LDHB*, *NR1D2*, and *PDK4* were downregulated. The expression determined by qRT-PCR was similar to that obtained by RNA-seq. Error bars in qRT-PCR indicate the standard error

To validate the RNA-seq results, we used quantitative RT-PCR (qRT-PCR) analysis to evaluate the two most significant hub genes (*EEF2* and *RPL7*) as potential biomarkers of AD for early diagnosis, three upregulated DEGs (*TRIOBP*, *TAS2R39*, and *BASP1*), and three downregulated DEGs (*LDHB*, *NR1D2*, and *PDK4*). Figure 7 summarizes the RNA-seq and qRT-PCR results. Although the 8 DEGs were not expressed at precisely the same levels in both RNA-seq and qRT-PCR analyses, the regulated trends of the 8 DEGs were entirely consistent (Fig. 7). These results demonstrated our RNA-seq data accurately estimates gene expression.

## Discussion

Peripheral blood biomarkers for early diagnosis have been examined in many diseases including AD [40–42]. In addition, various blood biomarkers associated with neurocognitive impairments have been reported, for example, glucose [43–45] and atherogenic index of plasma (AIP) [46]. However, no reliable and sensitive blood biomarkers are routinely used in clinical practice yet. One powerful and widely used approach to detect blood-based biomarkers, next-generation RNA-seq in human PBMCs, allows a comprehensive analysis of the entire transcriptome, but many of the previous studies were conducted in a small number of samples, particularly for AD.

Here, we performed comprehensive RNA-seq analysis using a large number of samples, to detect potential blood-based biomarkers associated with early diagnosis of AD. First, we used the bulk RNA-seq data to evaluate the difference in cell-type composition among AD, MCI, and CN samples. Of the 12 major immune cell types (B cells, plasma cells, CD8^+^ T cells, CD4^+^ T cells, γδ T cells, NK cells, monocytes, macrophages, dendritic cells, mast cells, eosinophils, and neutrophils), we found a statistically significant difference in the proportion of neutrophils; that is, an increase in the proportion of neutrophils was significantly associated with AD prognosis. In addition, the association of this increase with prognosis was further confirmed using a large number of additional samples obtained from routine blood tests. Although a recent report suggested that the neutrophil phenotype could be associated with the rate of cognitive decline and therefore might be a prognostic blood biomarker in patients with AD [47], the study involved only a few samples (*n* = 42). In contrast, our current results were obtained from not only different data sets (RNA-seq and routine blood tests) but also a far larger sample population (*n* = 3,099), providing stronger evidence that the proportion of neutrophils has the potential to be a blood biomarker of AD.

We also examined the DEGs between AD and CN samples. Of the 846 total statistically significant DEGs identified, 480 genes were upregulated and 366 were downregulated in AD. To gain further insight into the biological functions of the identified DEGs, we performed GSEA and PPI network analysis. Multiple statistically significant GO terms, one KEGG biological pathway, and several important hub genes were identified. A risk prediction model using the top two hub genes (*EEF2* and *RPL7*) and the proportion of neutrophils increased the model’s AUC, compared with that of a model using clinical information only. Therefore, our model provides an effective and precise prediction of AD risk.

One of the potential biomarkers, *EEF2*, is a member of the GTP-binding translation elongation factor family and an essential factor for protein synthesis and cell survival. In recent studies, *EEF2* kinase reduction alleviated AD-associated defects in AD model mice [48]. In addition, *RPL7* is reported to be a tau-dependent T cell intracellular antigen 1 (*TIA1*)-interacting protein [49, 50]. *TIA1* co-localizes with neuropathology in brain tissue of subjects with AD, frontotemporal lobar dementia, and amyotrophic lateral sclerosis, as well as in animal models of these diseases [51–53], all of which are associated with pathological tau misfolding and aggregation. These results suggest that these two hub genes could play a key role in the pathogenesis of AD.

We applied our risk prediction model—constructed by using these three potential biomarkers (proportion of neutrophils, *EEF2*, and *RPL7*) and three clinical features (age, sex, and *APOE* ε_4_ genotypes)—to prospective cohort data. Although the highest variable importance was age among the six features, the three potential biomarkers interestingly showed a higher variable importance than the other clinical features (age and *APOE* ε_4_ genotypes). In general, when a risk prediction model is constructed by using AD and CN samples, it is difficult to apply to MCI samples. However, because our prediction model provides a probability of AD conversion for each sample, we were able to make it applicable to MCI samples simply by adjusting the cutoff probability for conversion. Our risk prediction model significantly classified MCI samples into two categories (high and low risks) and yielded a high NPV of 0.848. For clinical use, this prospective prediction model must have high NPV because it likely will be used at the first screening for AD conversion. This risk prediction model requires further refinement before its practical use in healthcare. One improvement would be to consider genetic variations, such as single-nucleotide variants, short insertions and deletions, and copy number variations, because GWAS have revealed many types of genetic variation that contribute to AD risk [6–8] In addition, the combination of genetic variation and gene expression—expression quantitative trait loci (eQTLs) [54–56], which are genetic variants that affect gene expression levels—should be considered for the improvement of AD risk prediction models. Integration of that genetic variation, along with eQTL effects, likely will further improve the prospective AD risk prediction model.

## Conclusions

The current study identified potential biomarkers for early diagnosis of AD from RNA sequencing data. The risk prediction model constructed by using the biomarkers achieved a high AUC for a validation cohort; when further applied to a prospective cohort, the model achieved high accuracy. Our model was demonstrated to be effective in prospective AD risk prediction. These findings indicate the discovery of potential biomarkers for early diagnosis of AD, and their further improvement may lead to future practical clinical use.

## Supplementary information

**Additional file 1:.** Supplementary Table S1. Clinical information of samples used in RNA-seq data.

**Additional file 2:.** Supplementary Table S2. Clinical information of samples used in routine blood tests.

**Additional file 3:.** Supplementary Table S3. RNA-seq data.

**Additional file 4:.** Supplementary Table S4. A list of all detailed DEGs.

**Additional file 5: **Supplementary Table S5. TPM of *RPL7* and *EEF2*.

**Additional file 6:.** Supplementary Figure S1. Risk prediction models constructed using clinical information and two hub genes expression. The ROC curves of our risk prediction models in a test set. (a) AUC = 0.878 in AD and CN (b) AUC = 0.683 in MCI and CN (c) AUC = 0.645 in MCI and AD.

## Data Availability

All datasets used or analyzed in the current study are available from the corresponding author on reasonable request.
