# Supplementary material for: Identification of potential blood biomarkers for early diagnosis of Alzheimer’s disease through RNA sequencing analysis
Source: Alzheimers Res Ther. 2020 Jul 16;12:87. doi: 10.1186/s13195-020-00654-x (PMC7367375; doi:10.1186/s13195-020-00654-x)
Supplement: Supplementary file 6 — Additional file 6:. Supplementary Figure S1. Risk prediction models constructed using clinical information and two hub genes expression. The ROC curves of our risk prediction models in a test set. (a) AUC = 0.878 in AD and CN (b) AUC = 0.683 in MCI and CN (c) AUC = 0.645 in MCI and AD. [file 13195_2020_654_MOESM6_ESM.pdf]

Supplementary Figure S1. Risk prediction models constructed using clinical information and two hub genes expression

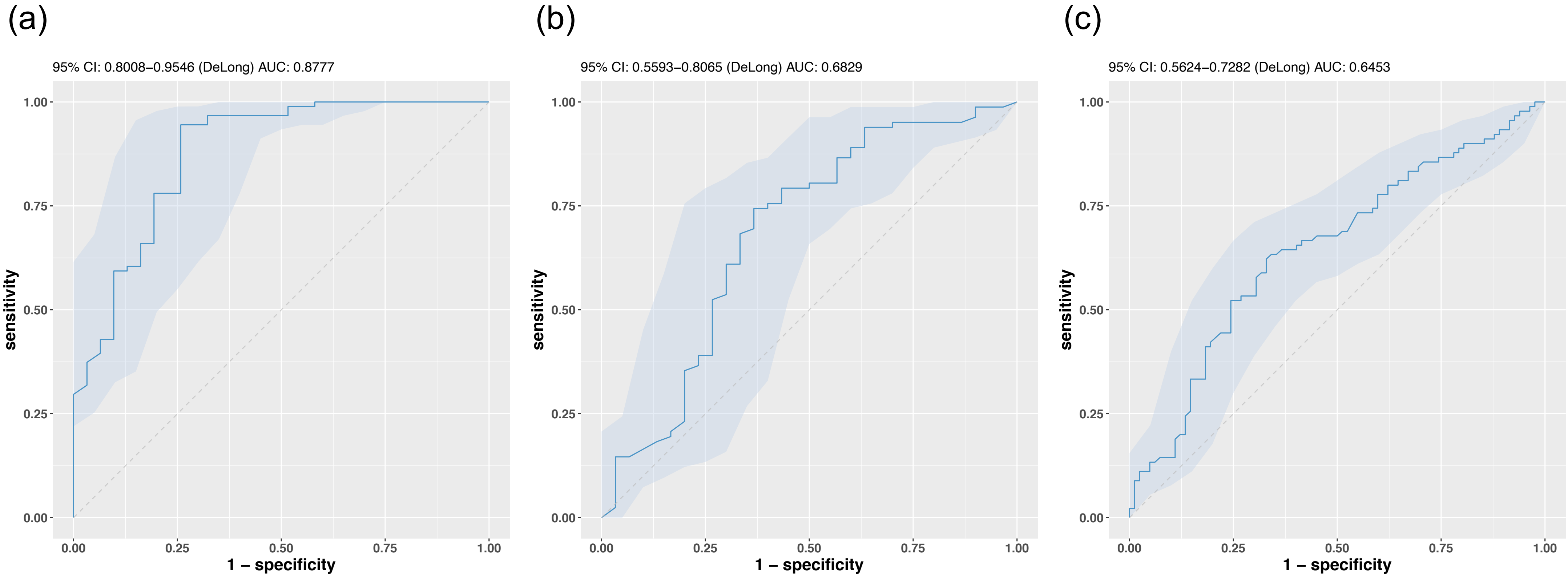

The ROC curves of our risk prediction models in a test set. (**a**) AUC = 0.878 in AD and CN (**b**) AUC = 0.683 in MCI and CN (**c**) AUC = 0.645 in MCI and AD.
